# Association between ABO and Rh blood groups and subtypes of age-related macular degeneration: A cross-sectional study

**DOI:** 10.1371/journal.pone.0354886

**Published:** 2026-07-28

**Authors:** Hasan Öncül, Umut Dağ, Mehmet Fuat Alakuş, Betül Dertsiz Kozan, Mehtap Savar Çağlayan

**Affiliations:** Department of Ophthalmology, University of Health Sciences Diyarbakır Gazi Yaşargil Training and Research Hospital, Diyarbakır, Türkiye; West Bengal University of Animal and Fishery Sciences, INDIA

## Abstract

Age-related macular degeneration (AMD) is a leading cause of irreversible visual impairment and is driven by complex genetic, inflammatory, and vascular mechanisms. ABO and Rh blood group antigens, which are expressed on vascular endothelium and involved in immune and hemostatic pathways, have been implicated in various systemic diseases; however, their potential association with AMD phenotypic variation remains unclear. In this cross-sectional study, 488 patients with clinically confirmed AMD from a tertiary referral center were included. AMD was classified as neovascular AMD (wet AMD) or nonneovascular AMD (dry AMD) based on multimodal imaging and standardized diagnostic criteria. ABO and Rh blood group data were obtained from medical records, and individuals with major known AMD risk factors, including smoking, alcohol consumption, and obesity, were excluded to reduce confounding. Associations between AMD subtypes and ABO blood groups and Rh factor were evaluated using the Pearson chi-square test, and crude odds ratios (ORs) with 95% confidence intervals (CIs) were calculated. The mean age of participants was 66.9 ± 4.7 years, and 273 patients (55.9%) had neovascular AMD. A statistically significant association was observed between ABO blood group distribution and AMD subtypes (χ² = 8.31, p = 0.040). A higher proportion of patients with neovascular AMD had blood group A, whereas blood group B was relatively more common among patients with nonneovascular AMD. However, individual pairwise crude odds ratio analyses did not demonstrate statistically significant associations for specific ABO blood groups. No association was observed for Rh status. These findings suggest a possible modest association between overall ABO blood group distribution and AMD phenotype; however, the clinical significance of this relationship remains uncertain, particularly in the absence of multivariable adjustment. Larger multicenter studies with comprehensive multivariable analyses are warranted.

## Introduction

Age-related macular degeneration (AMD) is a leading cause of irreversible visual impairment among older adults worldwide, affecting approximately 196 million individuals in 2020 and projected to reach 288 million by 2040 [1]. Both nonmodifiable and modifiable risk factors contribute to disease development, including advanced age, genetic predisposition, smoking, alcohol consumption, and increased body mass index [2,3]. The pathogenesis of AMD is multifactorial and involves complex interactions among oxidative stress, chronic inflammation, and choroidal vascular dysfunction [4].

Clinically, AMD is broadly classified into nonneovascular and neovascular forms, which differ in pathophysiology, progression, and visual outcomes, with the neovascular form accounting for the majority of severe vision loss [5,6].

The ABO blood group system is determined by carbohydrate antigens expressed not only on erythrocytes but also on vascular endothelial cells and various tissues [7,8]. Beyond their classical role in transfusion medicine, ABO antigens are involved in immune regulation, cell adhesion, and vascular homeostasis [7,9,10]. Systemic factors such as smoking, alcohol use, and metabolic status are also known to influence AMD development and progression [11,12].

Inflammatory and immune-mediated mechanisms play a central role in AMD pathogenesis [13,14]. Genetic studies have identified multiple susceptibility loci, particularly variants in complement-related genes and the ARMS2/HTRA1 region, which are strongly associated with disease susceptibility and progression [15–18]. In addition, retinal structural characteristics, including macular thickness, are influenced by genetic determinants, and emerging evidence suggests that these parameters may vary according to ABO and Rh blood group systems [19].

Molecular studies have further emphasized the role of dysregulation of the complement pathway as a key driver of AMD development [20–24]. Given that ABO and Rh blood group antigens are associated with inflammatory mediators, endothelial function, and microvascular processes, a relationship between blood group systems and AMD phenotypes is biologically and mechanistically plausible [25,26].

Despite this strong biological rationale, the relationship between ABO and Rh blood groups and AMD has not been specifically investigated in the context of disease subtypes. In particular, it remains unclear whether these blood group systems are associated with the distinction between neovascular and nonneovascular AMD. Addressing this gap may provide novel insight into the potential contribution of systemic factors to AMD heterogeneity.

Therefore, the aim of this study was to investigate the association between ABO and Rh blood group systems and AMD subtypes in a well-defined clinical cohort. We hypothesized that, given their roles in inflammatory and vascular pathways, blood group antigens may be associated with differences in AMD phenotype.

## Materials and methods

### Study design and participants

This cross-sectional study was conducted from 12 May 2025 to 31 December 2025 at the Retina Clinic of a tertiary referral center. A total of 488 consecutive patients with a clinical diagnosis of age-related macular degeneration (AMD) who were under regular follow-up were included. Demographic characteristics and medical history were obtained from structured interviews and electronic medical records. ABO and Rh blood group data were retrieved from official health records; when official records were unavailable, blood typing was performed using standard serological methods during clinical evaluation.

Information on smoking status and alcohol consumption was recorded. Body mass index (BMI) was calculated as weight in kilograms divided by height in meters squared (kg/m^2^). All data were independently entered and cross-checked to ensure accuracy.

The study was conducted in accordance with the Declaration of Helsinki and approved by the Ethics Committee of the University of Health Sciences Diyarbakır Gazi Yaşargil Training and Research Hospital (Approval No. 454). Written informed consent was obtained from all participants.

### Ophthalmological assessment and AMD classification

All participants underwent a comprehensive ophthalmological examination performed by two experienced ophthalmologists. Pupillary dilation was achieved using topical tropicamide. Anterior and posterior segment evaluations were performed using slit-lamp biomicroscopy with a + 90 diopter lens. Optical coherence tomography (OCT) imaging was performed using a spectral-domain device equipped with an eye-tracking system to minimize motion artifacts. AMD was classified into neovascular AMD and nonneovascular AMD based on multimodal assessment, including clinical examination, fundus evaluation, OCT imaging, and fundus fluorescein angiography when indicated. Nonneovascular AMD was defined by the presence of drusen, retinal pigment epithelium irregularities, or geographic atrophy in the absence of intraretinal or subretinal fluid or hemorrhage. Neovascular AMD was defined by the presence of subretinal or intraretinal fluid, hemorrhage, pigment epithelial detachment, or choroidal neovascularization.

### Inclusion and exclusion criteria

To reduce the influence of potential confounding factors, patients were recruited from the same geographic region. Because the study was exploratory and sample size constraints limited multivariable modeling, individuals with known major modifiable risk factors for AMD, including smoking, alcohol consumption, and obesity (BMI ≥ 30 kg/m^2^), were excluded a priori to reduce confounding. Patients who converted from nonneovascular to neovascular AMD during follow-up were also excluded to ensure stable subtype classification. This approach was adopted to reduce confounding; however, it resulted in a selected study population that may introduce selection bias.

### Statistical analysis

Statistical analyses were performed using IBM SPSS Statistics version 24 (IBM Corp., Armonk, NY, USA). Continuous variables were expressed as mean ± standard deviation, and categorical variables as frequencies and percentages. Associations between AMD subtypes and ABO blood groups and Rh factor were evaluated using the Pearson chi-square test. To further assess pairwise associations and effect size estimates, crude odds ratios (ORs) with 95% confidence intervals (CIs) were calculated for ABO blood groups using blood group O as the reference category and for Rh factor using Rh-negative status as the reference category. A two-tailed p-value of <0.05 was considered statistically significant. Analyses based on combined ABO/Rh phenotypes were not performed because several phenotype categories had small sample sizes, which could limit statistical reliability and render effect estimates unstable. Therefore, ABO blood groups and Rh status were analyzed separately.

## Results

A total of 488 patients with age-related macular degeneration (AMD) were included in the study. The mean age was 66.9 ± 4.7 years (range: 55–80), and 296 patients (60.7%) were male. Among the participants, 273 (55.9%) had neovascular AMD and 215 (44.1%) had nonneovascular AMD. The distribution of ABO and Rh blood groups is presented in Table 1. The most common blood groups were A Rh+ (33.4%) and O Rh+ (27.1%).

**Table 1:** Distribution of ABO and Rh blood groups in patients with AMD.

Blood group	n	%
A Rh+	163	33.4
A Rh−	23	4.7
B Rh+	73	15.0
B Rh−	9	1.8
AB Rh+	42	8.6
AB Rh−	8	1.6
O Rh+	132	27.1
O Rh−	38	7.8
Total	488	100

In Pearson chi-square analysis, a statistically significant association was observed between ABO blood group distribution and AMD subtypes (χ^2^ = 8.31, p = 0.040; Table 2). A higher proportion of patients with neovascular AMD had blood group A, whereas blood group B was relatively more common among patients with nonneovascular AMD.

**Table 2:** Association between ABO blood groups and AMD subtypes.

Blood group	Neovascular AMD (n, %)	Nonneovascular AMD (n, %)	Total (n, %)
A	113 (41.4)	73 (34.0)	186 (38.1)
B	37 (13.6)	45 (20.9)	82 (16.8)
AB	33 (12.1)	17 (7.9)	50 (10.2)
O	90 (33.0)	80 (37.2)	170 (34.8)
**Total**	**273 (100)**	**215 (100)**	**488 (100)**

No significant association was found between Rh factor and AMD subtype (χ^2^ = 0.346, p = 0.556; Table 3).

**Table 3:** Association between Rh factor and AMD subtypes.

Rh group	Neovascular AMD (n, %)	Nonneovascular AMD (n, %)	Total (n, %)
Rh+	227 (83.2)	183 (85.1)	410 (84.0)
Rh−	46 (16.8)	32 (14.9)	78 (16.0)
**Total**	**273 (100)**	**215 (100)**	**488 (100)**

To further evaluate these findings, crude odds ratios (ORs) with 95% confidence intervals (CIs) were calculated using blood group O as the reference category. Compared with blood group O, blood group A was associated with higher odds of neovascular AMD; however, this association did not reach statistical significance (OR: 1.38, 95% CI: 0.90–2.10). Blood group AB also demonstrated higher odds, but without statistical significance (OR: 1.73, 95% CI: 0.89–3.33). In contrast, blood group B was associated with lower odds of neovascular AMD, which was likewise not statistically significant (OR: 0.73, 95% CI: 0.43–1.24).

Regarding Rh status, Rh-positive individuals had slightly lower odds of neovascular AMD compared with Rh-negative individuals; however, this difference was not statistically significant (OR: 0.86, 95% CI: 0.53–1.41).

Although the overall ABO distribution differed significantly between AMD subtypes, all individual crude odds ratio confidence intervals crossed unity, indicating uncertainty regarding specific pairwise associations. No significant association was observed for Rh factor.

## Discussion

In this cross-sectional study of 488 patients with age-related macular degeneration (AMD), a statistically significant difference in overall ABO blood group distribution was observed between neovascular and nonneovascular AMD subtypes. Blood group A was more frequently represented among patients with neovascular AMD, whereas blood group B showed a relatively higher proportion among patients with nonneovascular AMD. However, individual pairwise crude odds ratio analyses using blood group O as the reference category did not demonstrate statistically significant associations, and no significant association was observed for Rh blood group status. These findings suggest that while ABO blood group distribution may show a modest relationship with AMD phenotype, the clinical significance of this association remains uncertain.

AMD is a complex and multifactorial disease involving interactions among genetic susceptibility, oxidative stress, inflammation, and vascular dysfunction [13,14]. Extensive evidence indicates that immune-mediated mechanisms, particularly complement pathway dysregulation, are central to AMD pathogenesis [22–24]. In addition, genetic variants such as those in the CFH and ARMS2/HTRA1 loci have been strongly associated with disease susceptibility and progression [20–21]. These well-established mechanisms likely exert a dominant influence, potentially overshadowing weaker or indirect contributions from systemic factors such as blood group antigens.

ABO blood group antigens are expressed not only on erythrocytes but also on vascular endothelial cells and have been implicated in inflammatory and thrombotic processes [7–10,25,26]. Previous studies have shown that ABO blood groups may influence circulating inflammatory mediators and endothelial function, supporting a potential role in diseases with vascular and inflammatory components [25,26]. Furthermore, emerging evidence suggests that retinal structural parameters, including macular thickness, may vary according to ABO and Rh blood group systems [19]. These observations provide a biologically and mechanistically plausible basis for investigating a potential relationship between blood group systems and AMD.

Despite this biological plausibility, an overall difference in ABO blood group distribution was observed between AMD subtypes; however, individual pairwise associations were not statistically significant. This suggests that if blood group-related effects exist, they are likely modest relative to the dominant genetic and inflammatory mechanisms underlying AMD. From a clinical perspective, the current findings do not support the routine use of ABO or Rh blood group status as biomarkers for distinguishing AMD subtypes.

Another important consideration is statistical power. Although the sample size in this study was relatively large, it may still have been insufficient to detect small subgroup-specific effects. In addition, the use of crude odds ratios without multivariable adjustment represents a methodological limitation, as residual confounding may persist despite the exclusion of major risk factors.

The modest association observed in overall ABO distribution may reflect indirect systemic influences rather than a strong mechanistic contribution to local retinal pathology. AMD pathophysiology primarily involves localized retinal and choroidal processes, including complement activation, immune cell infiltration, and structural degeneration [22–24]. Systemic factors such as blood group antigens may therefore exert only limited or indirect effects on these localized mechanisms.

This study has several strengths. To our knowledge, it is the first study specifically evaluating the association between ABO and Rh blood group systems and AMD subtypes. The relatively large sample size and standardized classification based on multimodal imaging enhance the internal validity of the findings. In addition, the exclusion of major modifiable risk factors was intended to reduce confounding.

Several limitations should be considered when interpreting these findings. First, the cross-sectional design precludes causal inference regarding the relationship between blood group systems and AMD subtype. Second, the study was conducted at a single tertiary referral center, which may limit generalizability. Third, no healthy control group was included, as the primary objective was comparison between AMD subtypes rather than disease susceptibility. In addition, multivariable adjustment was not performed. Although major modifiable AMD risk factors were excluded at the design stage to reduce confounding, residual confounding from other clinical variables such as age, sex, vascular comorbidities, and treatment-related factors cannot be excluded. Therefore, the observed associations should be interpreted cautiously, as independent effects could not be established without multivariable adjustment. Furthermore, detailed baseline subgroup characteristics were not consistently available for all participants, precluding presentation of a comprehensive comparison table between neovascular and nonneovascular AMD groups. This limitation restricts direct assessment of potential differences between the study groups and may limit evaluation of residual confounding. Furthermore, the exclusion of patients with major predefined risk factors and those with unstable AMD subtype classification resulted in a selected study population, which may reduce the external validity of the findings in routine clinical settings. While this design was intended to reduce confounding in an exploratory analysis, it may also have introduced selection bias. Finally, although the sample size was relatively large, it may still have been insufficient to detect small subgroup-specific effects.

Future studies should include larger, multicenter cohorts and incorporate comprehensive multivariable analyses. Integration of genetic, inflammatory, and molecular biomarkers may help clarify whether subtle interactions exist between blood group systems and the pathophysiology of AMD. Longitudinal designs may also provide insight into potential roles in disease progression rather than subtype differentiation.

In conclusion, this exploratory cross-sectional study observed a statistically significant difference in overall ABO blood group distribution between neovascular and nonneovascular AMD subtypes, whereas no association was observed for Rh status. However, the observed ABO association should be considered exploratory and descriptive. Individual pairwise associations were not statistically significant, and an independent association cannot be established from the current analysis because multivariable adjustment was not performed. Therefore, the clinical significance of the observed finding remains uncertain and requires confirmation in larger studies with comprehensive adjusted analyses. Larger multicenter studies with comprehensive adjusted analyses are needed to clarify whether an independent association exists.
